# Extent of FLAIR Hyperintense Vessels May Modify Treatment Effect of Thrombolysis: A *Post hoc* Analysis of the WAKE-UP Trial

**DOI:** 10.3389/fneur.2020.623881

**Published:** 2021-02-04

**Authors:** Anne Sophie Grosch, Anna Kufner, Florent Boutitie, Bastian Cheng, Martin Ebinger, Matthias Endres, Jochen B. Fiebach, Jens Fiehler, Alina Königsberg, Robin Lemmens, Keith W. Muir, Norbert Nighoghossian, Salvador Pedraza, Claus Z. Siemonsen, Vincent Thijs, Anke Wouters, Christian Gerloff, Götz Thomalla, Ivana Galinovic

**Affiliations:** ^1^Center for Stroke Research Berlin, Charité-Universitätsmedizin Berlin, Berlin, Germany; ^2^Klinik und Hochschulambulanz für Neurologie, Charité-Universitätsmedizin Berlin, Berlin, Germany; ^3^Berlin Institute of Health (BIH), Berlin, Germany; ^4^Hospices Civils de Lyon, Service de Biostatistique, Lyon, France; ^5^Université Lyon 1, Villeurbanne, France; ^6^Centre National de la Recherche Scientifique, Unité Mixte de Recherche 5558, Laboratoire de Biométrie et Biologie Evolutive, Equipe Biostatistique-Santé, Villeurbanne, France; ^7^Department of Neurology, Head and Neurocenter, University Medical Center Hamburg-Eppendorf, Hamburg, Germany; ^8^Department of Neurology, Medical Park Berlin Humboldtmühle, Berlin, Germany; ^9^German Centre for Cardiovascular Research (DZHK), Berlin, Germany; ^10^German Center for Neurodegenerative Diseases (DZNE), Berlin, Germany; ^11^Excellence Cluster NeuroCure, Charite-Universitätsmedizin Berlin, Berlin, Germany; ^12^Department of Diagnostic and Interventional Neuroradiology, University Medical Center Hamburg-Eppendorf, Hamburg, Germany; ^13^Department of Neurology, University Hospitals Leuven, Leuven, Belgium; ^14^Department of Neurosciences, Experimental Neurology, Katholieke Universiteit Leuven-University of Leuven, Leuven, Belgium; ^15^Laboratory of Neurobiology, Center for Brain & Disease Research, Flanders Institute for Biotechnology, Leuven, Belgium; ^16^Institute of Neuroscience & Psychology, University of Glasgow, Glasgow, United Kingdom; ^17^Department of Stroke Medicine, Claude Bernard University Lyon 1, CREATIS National Center for Scientific Research Mixed Unit of Research 5220-National Institute of Health and Medical Research U1206, National Institute of Applied Sciences of Lyon, Lyon Civil Hospices, Lyon, France; ^18^Department of Radiology, Girona Institute of Biomedical Research, Institute of Diagnostic Imaging, Dr. Josep Trueta Hospital, Girona, Spain; ^19^Department of Neurology, Aarhus University Hospital, Aarhus, Denmark; ^20^Stroke Theme, Florey Institute of Neuroscience and Mental Health, University of Melbourne, Heidelberg, VIC, Australia; ^21^Department of Neurology, Austin Health, Heidelberg, VIC, Australia

**Keywords:** ischemic stroke, FLAIR hyperintensities, thrombolysis, wake-up stroke, prognosis, MRI, hyperintense vessel

## Abstract

**Background and Aims:** Fluid-attenuated inversion recovery (FLAIR) hyperintense vessels (FHVs) on MRI are a radiological marker of vessel occlusion and indirect sign of collateral circulation. However, the clinical relevance is uncertain. We explored whether the extent of FHVs is associated with outcome and how FHVs modify treatment effect of thrombolysis in a subgroup of patients with confirmed unilateral vessel occlusion from the randomized controlled WAKE-UP trial.

**Methods:** One hundred sixty-five patients were analyzed. Two blinded raters independently assessed the presence and extent of FHVs (defined as the number of slices with visible FHV multiplied by FLAIR slice thickness). Patients were then separated into two groups to distinguish between few and extensive FHVs (dichotomization at the median <30 or ≥30).

**Results:** Here, 85% of all patients (*n* = 140) and 95% of middle cerebral artery (MCA) occlusion patients (*n* = 127) showed FHVs at baseline. Between MCA occlusion patients with few and extensive FHVs, no differences were identified in relative lesion growth (*p* = 0.971) and short-term [follow-up National Institutes of Health Stroke Scale (NIHSS) score; *p* = 0.342] or long-term functional recovery [modified Rankin Scale (mRS) <2 at 90 days poststroke; *p* = 0.607]. In linear regression analysis, baseline extent of FHV (defined as a continuous variable) was highly associated with volume of hypoperfused tissue (β = 2.161; 95% CI 0.96–3.36; *p* = 0.001). In multivariable regression analysis adjusted for treatment group, stroke severity, lesion volume, occlusion site, and recanalization, FHV did not modify functional recovery. However, in patients with few FHVs, the odds for good functional outcome (mRS) were increased in recombinant tissue plasminogen activator (rtPA) patients compared to those who received placebo [odds ratio (OR) = 5.3; 95% CI 1.2–24.0], whereas no apparent benefit was observed in patients with extensive FHVs (OR = 1.1; 95% CI 0.3–3.8), *p*-value for interaction was 0.11.

**Conclusion:** While the extent of FHVs on baseline did not alter the evolution of stroke in terms of lesion progression or functional recovery, it may modify treatment effect and should therefore be considered relevant additional information in those patients who are eligible for intravenous thrombolysis.

**Clinical Trial Registration:** Main trial (WAKE-UP): ClinicalTrials.gov, NCT01525290; and EudraCT, 2011-005906-32. Registered February 2, 2012.

## Introduction

The fluid-attenuated inversion recovery (FLAIR) hyperintense vessel (FHV) sign is commonly observed on magnetic resonance imaging (MRI) of acute ischemic stroke patients and is represented by ipsilateral linear or serpentine hyperintensities on FLAIR sequences distal to the vessel occlusion (1–6). FHVs have been shown to be an independent predictor of large vessel occlusion. However, studies investigating the underlying pathophysiology and prognostic value of FHVs have yielded contradictory results (7).

While some have shown that FHVs are associated with increased collateralization, decreased lesion growth, and improved long-term functional recovery (6, 8–11), others have shown that patients with extensive FHVs have increased lesion growth and worse functional outcome 3 months poststroke (2, 4, 12, 13). The apparent discrepancies in previous studies regarding the diagnostic and prognostic value of FHVs may be due the use of different methodologies in the assessment of FHVs and inhomogeneous cohorts of patients in terms of treatment in the acute setting and time to MRI.

The aim of the present study was to investigate whether the extent of FHVs has an effect on stroke evolution in terms of lesion progression and long-term functional recovery in a cohort of acute ischemic stroke patients with middle cerebral artery (MCA) occlusion and unknown time of onset from the randomized controlled WAKE-UP trial (14). Furthermore, we investigated whether the extent of FHVs on baseline imaging modifies the treatment effect of thrombolysis and recanalization rates on follow-up imaging.

## Methods

### Patients

This is a retrospective study including patients who were enrolled in the multicenter, randomized, double-blind, placebo-controlled WAKE-UP trial (14). Trial patients were randomized to either treatment with alteplase or placebo. For this analysis, 165 patients with confirmed, unambiguous, unilateral, and single-vessel occlusion on time-of-flight magnetic resonance angiography (MRA-TOF) were included. Patients were excluded from final analysis if baseline FLAIR was not available or not ratable due to poor image quality.

### Clinical Assessment

Demographic data included age, gender, and presence or previous history of the following cardiovascular risk factors: smoking, alcohol consumption, arterial hypertension, atrial fibrillation, hypercholesterolemia, diabetes mellitus type II, coagulation disorder, transient ischemic attack, ischemic stroke, and/or intracranial hemorrhage. Clinical assessment comprised the National Institutes of Health Stroke Scale (NIHSS) on admission and follow-up (5–9 days poststroke, or if this data point was not available 22 to 36 h poststroke, considered short-term outcome in our analysis) as well as good long-term outcome defined as modified Rankin Scale (mRS) <2 at 90 days poststroke.

### Radiological Assessment

A central image-reading committee reviewed all images acquired for patient enrollment in the WAKE-UP trial and reevaluated imaging inclusion/exclusion criteria assessed by local investigators. A detailed description of image assessment within the trial (i.e., measurement of lesion volumes) has been previously published (14). For the current analysis, all acquired images were retrospectively reevaluated by two independent raters (ASG and IG) at the Center for Stroke Research Berlin at Charite University Hospital Berlin. In this subsample of the WAKE-UP trial, diffusion-weighted imaging (DWI) and FLAIR were available for all patients (*n* = 165) on hospital admission and in 154 patients (93%) at follow-up (22–36 h after hospital admission). Lesion volumes were derived from baseline and follow-up DWI imaging to determine relative (follow-up divided by baseline DWI lesion volume) and absolute lesion growth (follow-up subtracted by baseline DWI lesion volume).

We also assessed the evolution of FHVs from baseline to follow-up FLAIR. We defined that a reduction in FHVs was present if there was a drop of more than one slice affected by FHVs between baseline and follow-up imaging. Dynamic susceptibility contrast perfusion MRI [perfusion-weighted imaging (PWI)] of diagnostic quality was available in 66 of all patients (40%), and volumes of hypoperfusion were calculated using RAPID (https://www.rapidai.com) with a threshold of Tmax >6 s. PWI–DWI mismatch was defined as an absolute mismatch volume of >10 ml and a mismatch ratio between PWI and DWI of >1.2. Occlusion site was evaluated on MRA-TOF. For MCA occlusion analyses, we only included the occlusions sites ICA+M1, ICA+M2, and M3/M4. Recanalization status was classified into either complete or no/partial recanalization on follow-up compared to baseline imaging.

### Assessment of FLAIR Hyperintense Vessels

**Figure 1:**
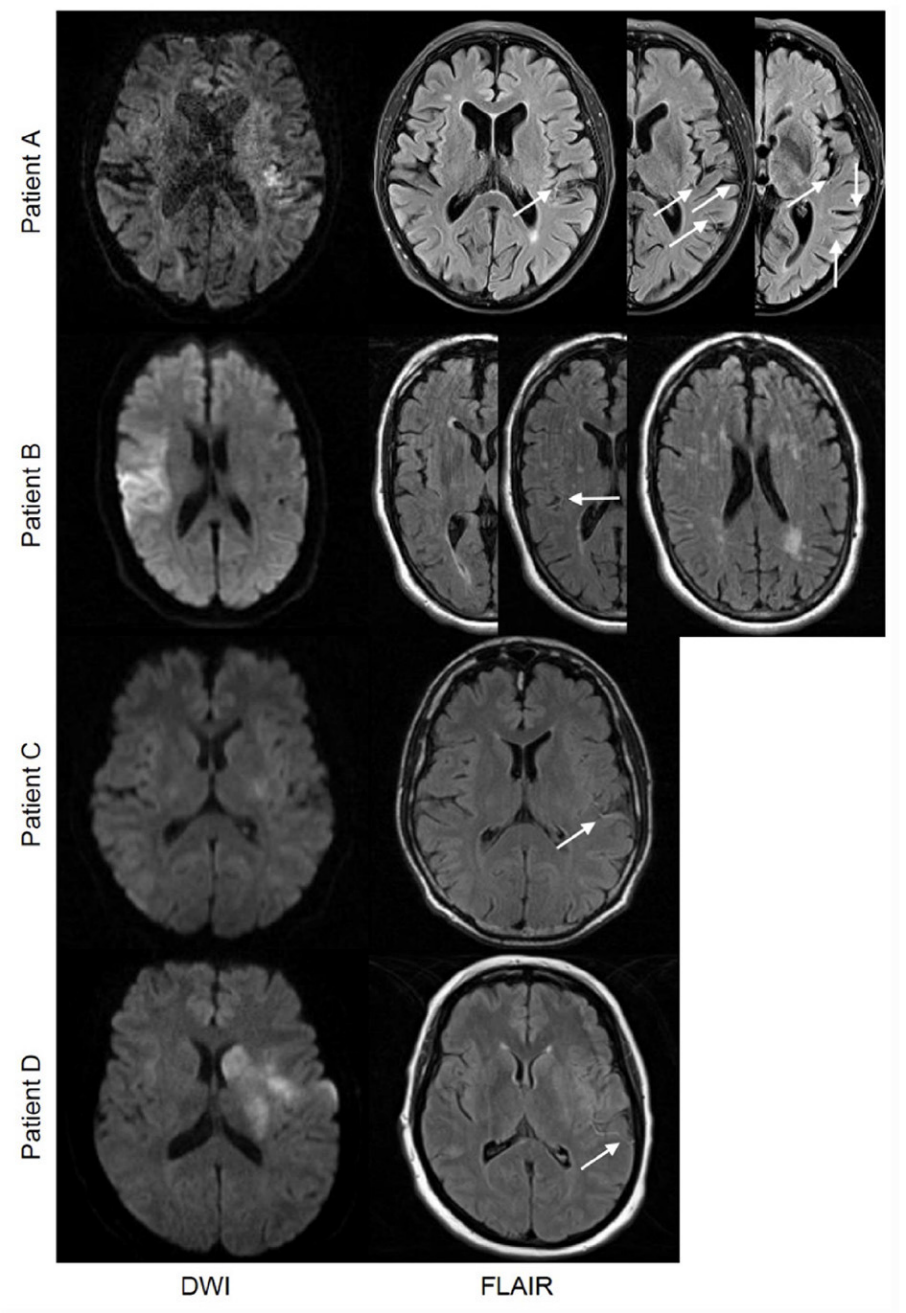
Patient A and Patient B represent two cases at different ends of the spectrum of the extent of FLAIR hyperintense vessels (FHVs). Patient A is a 69-year-old female with a left-sided M2 branch occlusion and baseline FHV extent of 60 (multiple linear and serpentine vessels visible surrounding the operculum and temporal lobe on all three images). Patient B is a 70-year-old male with a right-sided occlusion in the M2 branch of the middle cerebral artery whose initial extent of FHVs at baseline was 13 (a single serpentine vessel is visible between the operculum and the temporal lobe on the middle image). Both were treated with placebo; the modified Rankin Scale (mRS) at 90 days was 0 for patient A and 3 for patient B. Patients C and D represent cases with comparable FHV patterns but different stroke extent and severity at baseline. A comparison of two patients, one a 44-year-old male (patient C) and the other a 46-year-old female (patient D), both with a left-sided occlusion of the mainstem middle cerebral artery (MCA). The baseline extent of FHVs was 30 for both cases with a comparable distribution of vessels, yet the stroke volumes and distributions were different. Patient C showed only small scattered lesions in the insula, tip of the putamen, as well as the temporal and parietal lobes (total volume of 3 ml), while patient D showed an infarction encompassing the entire putamen and nucleus caudatus as well as portions of the insula and operculum, with additionally some scattered lesions in the frontal and parietal lobes (total volume of 15 ml). Their baseline National Institutes of Health Stroke Scale (NIHSS) score was also different (6 for patient C and 20 for patient D). At follow-up, both patients recanalized [patient C received recombinant tissue plasminogen activator (rtPA) and patient D received placebo]. They had a similar dynamics of FHVs showing a reduction in their extent (a complete reduction to zero in patient C and a partial reduction to 12 in patient D). Their mRS outcome at 90 days was 1 for patient C and 3 for patient D.

Blinded to clinical and radiological outcomes, two raters (ASG and IG) independently rated baseline and follow-up FLAIR images for the presence and extent of FHVs. FHVs were defined as linear or serpentine hyperintensities distal to the site of the occluded vessel (Figure 1). Due to different FLAIR slice thicknesses of the participating medical centers, the extent of FHVs was defined as the number of slices with visible FHVs multiplied by FLAIR slice thickness. Inter-rater agreement for the presence of FHVs was 95.76% with a free marginal kappa of 0.92 [95% confidence interval (CI) 0.85–0.98] at baseline and 88.49% with a free marginal kappa of 0.77 (95% CI 0.67–0.87) at follow-up. The two raters agreed on the extent of FHVs (up to a maximum difference of one slice) in 52% of all cases. Consensus was reached for discrepant cases. For further analysis, only patients with MCA occlusion were separated into two groups to distinguish between few and extensive FHVs (dichotomization at the median <30 or ≥30) (1, 2).

### Statistical Analysis

Spearman's rank correlation coefficient was used for correlation analyses. Based on the scale level of the variables, Mann–Whitney-U test, Fisher's exact test, or chi-square test were applied for two-group analyses. Binary logistic regression analyses were performed for recanalization (adjustment for reduction in FHVs, treatment group, and age) as well as for good outcome defined as mRS <2 at 90 days poststroke (adjustment for well-known predictors of outcome including baseline NIHSS score, recanalization status, treatment group, baseline lesion volume, occlusion site, FHV group, hours from last seen well to treatment). Linear regression analysis was performed for volume of hypoperfused tissue (adjustment for baseline extent of FHVs) as well as NIHSS score at follow-up (adjustment for baseline NIHSS score, recanalization status, treatment group, baseline lesion volume, occlusion site, FHV group). To investigate the interaction between the extent of FHVs and treatment effect on the primary endpoint, we used an unconditional logistic regression model, relating the log-odds of the primary outcome with the covariate of interest, the treatment group, and their interaction, with adjustment on NIHSS score at baseline. The interaction term was tested with the Wald–chi-square test, and the treatment effect [odds ratio (OR)] and its 95% CI were estimated for each category. Statistical analysis was performed using IBM SPSS (www.ibm.com, version 24) and *p* ≤ 0.05 were considered significant.

## Results

### Entire Patient Cohort

Out of 503 patients enrolled in the WAKE-UP trial, 165 met all inclusion criteria (328 were excluded due to absence of vessel occlusion, two due to poor image quality, three due to bilateral vessel occlusion, five due to unavailable imaging data). The mean age of this subgroup of patients was 64.2 years, 47% were female, median NIHSS score at baseline was 9.0 [interquartile range (IQR) 6.0–15.0]. In total, 85% (*n* = 140) had FHVs visible on baseline FLAIR, and median extent of FHVs was 30.0 (IQR 21.3–39.0). Of the 25 patients without baseline FHVs, four had an occlusion of the internal carotid artery (ICA) (16%), three of M2 or ICA+M2 (12%), four of M3 or M4 (16%), seven of the posterior cerebral artery (PCA) (28%), and seven of other vessels (28%).

### Patients With Middle Cerebral Artery Occlusion

In patients with MCA occlusion (*n* = 134, 81%), 95% had FHVs at baseline (*n* = 127), and the median extent of FHVs was 30.0 (IQR 24.0–40.0). Patients with extensive FHVs did not differ from patients with few FHVs in terms of baseline DWI lesion volumes (9.7 vs. 17.5 ml; *p* = 0.218) and baseline NIHSS scores (12.0 vs. 9.0; *p* = 0.147). Baseline extent of FHVs (defined as a continuous variable) was highly associated with the volume of hypoperfused tissue (β = 2.161; 95% CI 0.96–3.36; *p* = 0.001), with patients with extensive FHVs having significantly larger hypoperfused areas at baseline. The occlusion site also differed significantly between few and extensive FHVs, with extensive FHVs being associated with proximal vessel occlusions (*p* <0.001). Patients with few and extensive FHVs revealed no differences in the time between last seen well to MRI (*p* = 0.261), last seen well to treatment (*p* = 0.301), and MRI to treatment (*p* = 0.271). Likewise, continuous extent of FHVs did not correlate with any of the abovementioned variables. In terms of outcome, there were no differences in relative lesion growth (*p* = 0.971) or short-term (*p* = 0.342) or long-term functional recovery (*p* = 0.607) between groups (Table 1).

**Table 1:** Demographic data, baseline and follow-up clinical and radiological data for all patients, MCA occlusion patients, MCA occlusion patients with few FHVs, and MCA occlusion patients with extensive FHVs.

	**All patients (*n* = 165)**	**MCA occlusion patients (*n* = 134)**	**MCA occlusion patients with few FHVs (*n* = 53)**	**MCA occlusion patients with extensive FHVs (*n* = 74)**	***P*-value few vs. extensive FHVs**
Age, mean (SD)	64.2 (11.9)	64.5 (11.7)	63.9 (11.6)	64.9 (11.9)	0.514
Female sex, % (*n*)	47% (77)	49% (66)	38% (20)	54% (41)	**0.049**
**Previous history of CVRF, % (*****n*****)**					
-Arterial hypertension	49% (80)	49% (65)	48% (25)	48% (35)	0.988
-Atrial fibrillation	17% (27)	19% (25)	9% (5)	25% (18)	**0.036**
-TIA	3% (5)	3% (4)	6% (3)	1% (1)	0.307
-Ischemic stroke	10% (16)	9% (12)	9% (5)	7% (5)	0.741
-Intracranial hemorrhage	0% (0)	0% (0)	0% (0)	0% (0)	1.000
-Hypercholesterolemia	36% (56)	37% (47)	42% (21)	33% (23)	0.306
-Diabetes mellitus type II	13% (21)	15% (19)	15% (8)	13% (9)	0.792
-Coagulation disorder	0% (0)	0% (0)	0% (0)	0% (0)	1.000
-Gastrointestinal bleeding	2% (3)	2% (3)	6% (3)	0% (0)	0.066
-Current smoking	31% (48)	31% (40)	22% (11)	38% (27)	0.080
-Alcohol	39% (59)	41% (52)	41% (20)	44% (31)	0.707
**FHV on admission**					
-% (*n*)	85% (140)	95% (127)	100% (53)	100% (74)	1.000
-Median extent (IQR)	30.0 (21.3–39.0)	30.0 (24.0–40.0)	22.0 (17.5–25.0)	39.0 (30.0–48.0)	** <0.001**
**Reduction in FHVs between baseline and follow-up imaging**					
-Absolute, median (IQR)	15.0 (5.0–25.0)	15.0 (5.0–25.0)	14.0 (2.7-24.0)	15.0 (6.0-30.0)	0.059
-Relative, median (IQR)	50% (15%−100%)	50% (16%−100%)	100% (14%−100%)	40% (16%−83%)	**0.040**
ASPECTS mismatch, % (*n*)	73% (97)	73% (93)	59% (31)	84% (62)	**0.002**
**NIHSS score**					
-Baseline, median (IQR)	9.0 (6.0–15.0)	10.0 (6.0–15.5)	9.0 (6.0–14.0)	12.0 (7.0–16.0)	0.147
-Follow-up, median (IQR)	6.0 (2.0–13.3)	6.0 (1.5–13.0)	6.0 (1.0–10.0)	8.0 (2.0–15.0)	0.342
**MRS at 90 days**					
-Median (IQR)	3.0 (1.0–4.0)	3.0 (1.0–4.0)	3.0 (1.0–3.0)	3.0 (1.0–4.0)	0.255
-Good outcome, % (*n*)	26% (42)	27% (36)	39% (16)	26% (19)	0.607
**DWI lesion volume in ml**					
-Baseline, median (IQR)	9.8 (3.1–24.0)	10.4 (4.7–25.9)	17.5 (4.8–31.8)	9.7 (4.4–22.1)	0.218
-Follow-up, median (IQR)	21.9 (5.6–59.0)	23.1 (6.2–57.6)	29.7 (5.7–60.0)	21.3 (6.1–54.7)	0.653
**DWI lesion growth in %**					
-Absolute, median (IQR)	10.6 (1.1–38.3)	12.2 (1.9–36.2)	11.8 (1.7–36.2)	12.8 (1.6–37.0)	0.746
-Relative, median (IQR)	121% (37–320%)	121% (28–254%)	114% (14–253%)	121% (31–276%)	0.971
Treatment with rtPA, % (*n*)	50% (83)	50% (67)	57% (30)	45% (33)	0.186
Occlusion site, % (*n*)					** <0.001**
-ICA	6% (9)	0% (0)	0% (0)	0% (0)	
-ICA+M1 or M1	39% (64)	48% (64)	28% (15)	66% (49)	
-ICA+M2 or M2	27% (45)	34% (45)	45% (24)	24% (18)	
-M3/M4	15% (25)	19% (25)	26% (14)	10% (7)	
-PCA	9% (14)	0% (0)	0% (0)	0% (0)	
-Other	5% (8)	0% (0)	0% (0)	0% (0)	
Recanalization, % (*n*)	36% (49)	41% (45)	46% (19)	34% (21)	0.204
PWI-DWI mismatch, % (*n*)	65% (42)	78% (38)	72% (18)	83% (19)	0.499
PWI volume, median (IQR)	50.7 (26.0–89.7)	64.4 (30.2–95.1)	49.0 (29.3–72.3)	74.8 (50.3–109.2)	**0.047**
Hours from LSW to MRI, median (IQR)	10.0 (6.8–11.8)	10.1 (6.9–11.9)	10.1 (6.3–11.5)	10.2 (7.3–12.9)	0.261
Hours from LSW to treatment, median (IQR)	10.5 (7.4–12.4)	10.6 (7.5–12.5)	10.5 (6.9–12.1)	10.7 (7.6–13.4)	0.301
Hours from MRI to treatment, median (IQR)	0.4 (0.3–0.6)	0.4 (0.3–0.6)	0.4 (0.2–0.6)	0.5 (0.3–0.6)	0.271

*P-values are given for group comparisons between patients with few and extensive FHVs*.

*MCA, middle cerebral artery; FHV, FLAIR hyperintense vessel; SD, standard deviation; n, number; CVRF, cardiovascular risk factors; IQR, interquartile range; ASPECTS, Alberta stroke program early CT score; NIHSS, National Institutes of Health Stroke Scale; mRS, modified Rankin Scale; DWI, diffusion-weighted imaging; rtPA, recombinant tissue plasminogen activator; ICA, internal carotid artery; M1, M1 segment of the MCA; M2, M2 segment of the MCA, M3/M4, M3 or M4 segment of the MCA; PCA, posterior cerebral artery; PWI, perfusion-weighted imaging; LSW, last seen well. The bold values indicate the statistical significance (i.e., p <0.05)*.

### Middle Cerebral Artery Occlusion Patients: Functional Recovery and Treatment Effect

Univariate regression analysis of long-term functional recovery revealed merely baseline NIHSS score and recanalization as predictors. Treatment group, baseline DWI lesion volume, occlusion site, dichotomized extent of FHVs, and hours from last seen well to treatment were not identified as independent predictors in this subgroup analysis. Multivariable regression analysis confirmed baseline NIHSS score and recanalization as independent predictors for long-term functional recovery (Table 2).

**Table 2:** Univariate and multivariable regression analyses for good outcome (mRS <2) 3 months poststroke in MCA occlusion patients.

	**Univariable logistic regression**		**Multivariable logistic regression**	
	**Crude odds ratio (95% CI)**	***P*-value**	**Adjusted odds ratio (95% CI)**	***P*-value**
Baseline NIHSS score	0.768 (0.687; 0.858)	** <0.001**	0.753 (0.647; 0.878)	** <0.001**
Recanalization	3.873 (1.578; 9.508)	**0.003**	3.922 (1.147; 13.404)	**0.029**
Treatment group	1.618 (0.746; 3.506)	0.223	1.948 (0.584; 6.494)	0.278
Small baseline DWI lesion volume	0.971 (0.942; 1.000)	0.051	1.000 (0.958; 1.043)	0.988
Occlusion site (more distal)	1.405 (0.856; 2.306)	0.178	0.665 (0.272; 1.626)	0.371
FHV group (few vs. extensive)	0.814 (0.371; 1.785)	0.607	1.123 (0.308; 4.091)	0.861
Hours from LSW to treatment	0.973 (0.897; 1.054)	0.498	1.039 (0.922; 1.170)	0.528

*NIHSS, National Institutes of Health Stroke Scale; 95% CI, 95% confidence interval; DWI, diffusion-weighted imaging; FHV, FLAIR hyperintense vessel; mRS, modified Rankin Scale; LSW, last seen well; MCA, middle cerebral artery. The bold values indicate the statistical significance (i.e., p <0.05)*.

**Figure 2:**
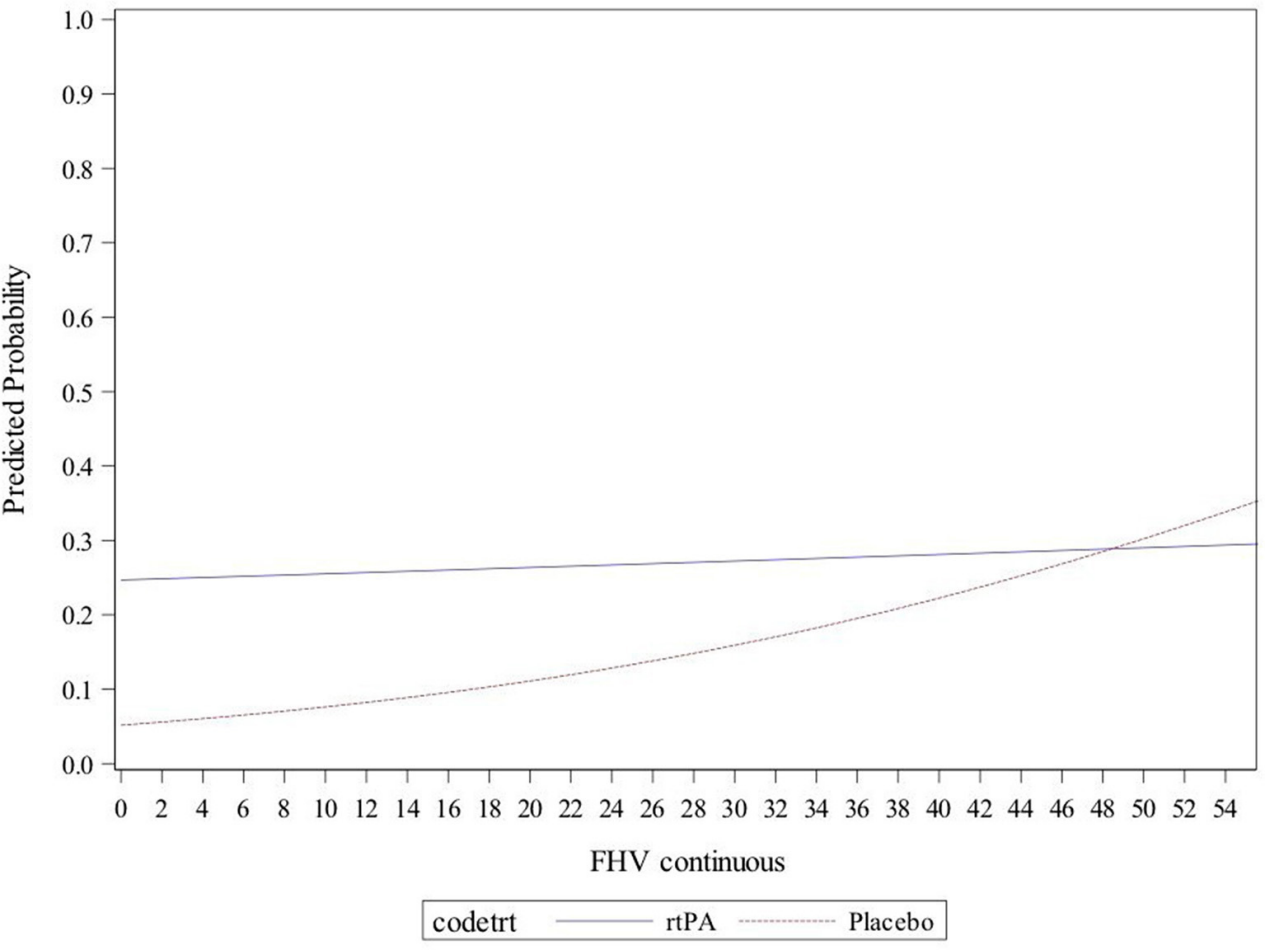
Predicted probability of good clinical outcome [modified Rankin Scale (mRS) <2] in patients grouped according to treatment, plotted against the extent of FLAIR hyperintense vessels (FHVs) on baseline imaging. The continuous blue line represents recombinant tissue plasminogen activator (rtPA)-treated patients, and the dotted red line stands for patients who received placebo.

When patients were separated into groups based on treatment, there was a clear trend pointing to the extent of FHVs as a factor that modifies treatment effect. In patients with FHV extent <30, only 14% of individuals with a proximal occlusion (M1 segment of the MCA) and 10% with a more distal occlusion (M2, M3, or M4 segments of the MCA) had good outcome if treated with placebo, whereas 25 and 46% of patients (with proximal and distal occlusions, respectively), had good outcome if given recombinant tissue plasminogen activator (rtPA). Accordingly, in patients with FHV extent <30, the odds for good outcome were increased by 5.3 in rtPA-treated patients as compared to those treated with placebo (OR = 5.3; 95% CI 1.2–24.0), whereas no apparent benefit of rtPA was observed in patients with FHV extent ≥30 (OR = 1.1; 95% CI 0.3–3.8), *p*-value for interaction = 0.11. There were no differences in baseline clinical or radiological parameters (including occlusion site) between patients who received placebo and those who received rtPA. When the extent of FHVs was treated as a continuous variable in tPA-treated patients, the probability of good outcome was relatively stable across the entire range of FHVs. However, in patients receiving placebo, there was a very low likelihood of a good outcome with less prominent FHVs, with chances improving parallel to increasing FHV extent (Figure 2).

### Recanalization and Reduction in FLAIR Hyperintense Vessels

Overall, the majority of patients (64%; *n* = 82) experienced a reduction in FHVs between baseline and follow-up; the median relative reduction was 50% (ICR 15–100%). In MCA occlusion patients, the relative extent of reduction was significantly more pronounced in patients who recanalized as compared to non-recanalizers (86 vs. 31%; *p* = 0.001). In binary logistic regression of MCA occlusion patients, a reduction in FHVs had an adjusted OR of 5.82 (adjusted for treatment group and age; 95% CI 2.00–16.92; *p* = 0.001) for successful recanalization on follow-up. There were only five patients who recanalized but did not show a reduction in FHVs on follow-up, whereas 33 patients showed a reduction in FHVs despite persistent vessel occlusion. Among these non-recanalizers, there was no difference in terms of absolute lesion progression (21.0 vs. 13.1 ml; *p* = 0.589), follow-up NIHSS score (9.0 vs. 7.0; *p* = 0.917), or 3-month mRS (3.0 vs. 3.0; *p* = 0.497) between patients who showed a reduction in FHVs and those who did not (Figure 1).

## Discussion

In the current study, the extent of FHVs on baseline imaging did not alter stroke progression in terms of initial stroke severity, lesion growth, or long-term functional recovery in patients with MCA occlusion and unknown time of symptom onset. However, patients with less pronounced FHVs had higher odds of achieving a good outcome following treatment with rtPA. In other words, the extent of FHVs assessed on acute imaging may modify the treatment effect of thrombolysis.

In line with previous studies (2, 4, 11), here, 85% of ischemic stroke patients with proven vessel occlusion presented with FHVs ipsilateral to the ischemic lesion on baseline imaging. Extent of FHVs correlated directly with the volume of hypoperfused tissue. This is likely in part due to the higher rates of proximal occlusions observed in patients with extensive FHVs (Table 1). Similar results were previously reported, showing an association between FHVs and more severe hypoperfusion (2) and identifying FHV as an independent predictor of a perfusion–diffusion mismatch in the case of vessel occlusion (15, 16).

**Figure 3:**
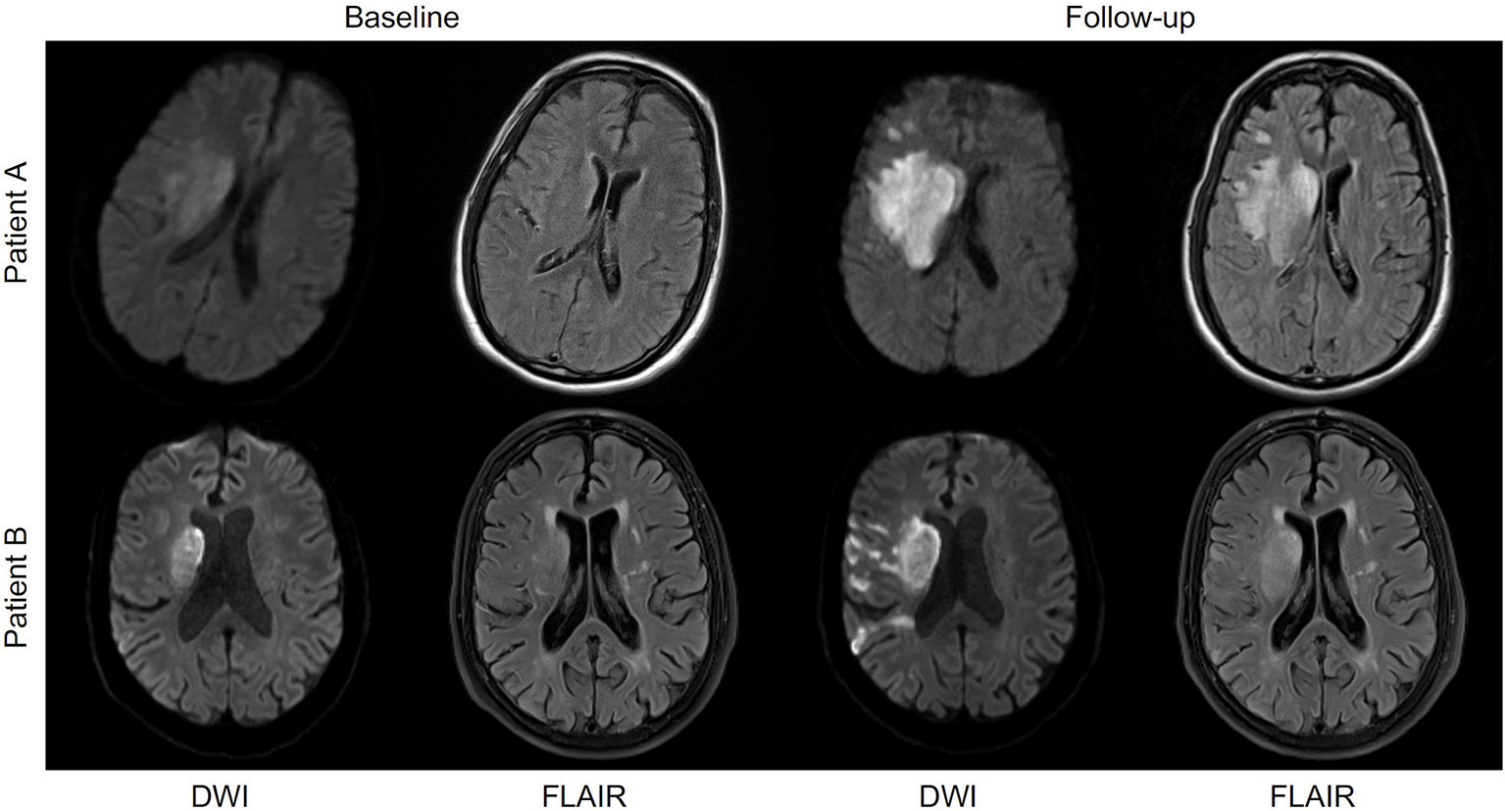
Patients A and B represent cases with comparable stroke evolution yet differing evolution of FLAIR hyperintense vessels (FHVs) between baseline and follow-up imaging. Both 64-year-old males with a right-sided M1 occlusion. Patient A additionally had an occlusion of the ipsilateral internal carotid artery (ICA). The baseline lesions were comparable in terms of volume and pattern (predominantly basal ganglia involvement and lesion size up to 20 ml). Their baseline National Institutes of Health Stroke Scale (NIHSS) score was also comparable (15 for patient A and 14 for patient B). The baseline extent of FHVs was different between the patients (30 in patient A and 50 in patient B). Both patients received placebo, and neither of them experienced a recanalization by the time of follow-up. There was a pronounced difference in the dynamics of FHVs between the patients, with patient A showing no more visible FHVs at follow-up, whereas the extent of FHVs in patient B actually increased from 50 to 55. Their outcome, however, was the same (both had a 90-day mRS of 4), and their final lesion volumes were almost identical (patient A 52 ml and patient B 59 ml).

In our study, the extent of FHVs had no effect on clinical stroke severity or lesion size on admission, nor did it modify lesion progression or functional recovery (Table 1). This matches the results of a recently published systematic review of FHVs in ischemic stroke (7); in a pooled sample of over 3,000 patients, there was no association between functional outcome and extent of FHVs. To further illustrate this, in our cohort, we found examples of patients with matching occlusions, similar lesion extent, and severity of stroke who presented with very different extents of FHVs at baseline as well as the opposite (patients with identical occlusions and similarly pronounced FHVs yet different clinical and imaging stroke severities) (Figure 3).

Interestingly, the extent of baseline FHVs modified treatment effect, with thrombolysis being more effective in patients with fewer visible collaterals, and especially so if they had a more distally placed vessel occlusion. Although patients with large vessel occlusions still benefit from intravenous thrombolysis, previous studies have shown that the presence of a proximally placed vessel occlusion is associated with worse outcome following intravenous thrombolysis (17) (additional REF). At the same time, for patients receiving placebo, higher likelihoods of good clinical outcome were found in individuals with more pronounced FHVs (Figure 2). This might point to a protective component of prominent FHVs, at least in the initial hours after occlusion occurs, with patients who are unable to quickly recruit an extensive collateral network being that much more dependent on therapy for a chance at good functional outcome. The generalizability of these results to different patient populations, i.e., to ischemic stroke patients with large vessel occlusion eligible for endovascular therapy should be viewed with caution. According to the clinical and radiological criteria of the DAWN and DEFUSE 3 trials, these patients would be candidates for direct endovascular therapy (18, 19). However, a better understanding of rtPA efficacy in patients with unknown symptom onset and extensive FHVs could be particularly valuable in selecting patients who might benefit from a bridging therapy with rtPA before endovascular therapy. Larger independent cohort analyses on this topic are warranted to validate our findings.

In this study, treatment with tPA did not reach statistical significance for good outcome 90 days poststroke in the overall cohort (Table 2). This is most likely due to the smaller sample size of the current study; point estimates for treatment were similar in this analysis (crude OR of 1.62) to those reported in the original trial analysis (crude OR 1.6) (14).

It is known that FHVs are a transient MRI phenomenon and typically disappear by 36 h poststroke (5, 20, 21). Similar to previous studies, we observed an overall reduction of FHVs over time in ~64% of patients (5, 22), and this reduction was independently associated with successful recanalization. In other words, early reduction in FHVs may be a surrogate marker of successful recanalization and hence be associated with less stroke progress and better functional recovery. However, in the case of persistent vessel occlusion, a reduction in FHVs was not associated with a smaller lesion growth or better functional recovery (Figure 1).

Interestingly, there were significantly more females in the group of patients with MCA occlusion and extensive FHVs (Table 1). Previous studies have described sex-specific differences in cerebrovascular parenchymal hyperintensities on FLAIR (23) (additional REF). However, to the best of our knowledge, previous studies on FHVs have not observed sex-specific differences in terms of the extent of FHVs in the setting of acute stroke. Future analyses on this topic would be of great interest.

Based on previous studies and our current analysis, it is clear that FHVs are radiological markers of proximal vessel occlusion. They most likely represent arteries distal to the occlusion site exhibiting slow flow (owing to a collateral circulation that is sufficient enough to provide retrograde flow but insufficient to achieve the extent of perfusion present prior to the stroke and therefore associated with the size of the perfusion deficit (2, 5, 12). This, however, is not to say that FHVs indicate hypoperfusion below the ischemia threshold leading to tissue infarction, as their presence and magnitude seem to confer a certain protective advantage to the tissue—an advantage that, as many studies have shown (7), is neither unequivocal nor easy to understand. In this they are not alone, as other MRI markers (for example, dynamic susceptibility contrast MRI, also known as perfusion imaging) have failed to deliver an indisputable parameter and/or threshold that reliably predicts tissue fate (24). The reason might lie in the highly dynamic evolution of an acute ischemic stroke; the timely unfolding of several factors, such as treatment, changes in antegrade flow (the extent of recanalization), and retrograde flow (the continuous improvement of collateral circulation), but also different tissue susceptibilities to ischemia collectively play crucial roles in determining tissue fate. Therefore, any given MRI must be seen as a snapshot of the current situation, which is inevitably destined to undergo change and can hence only partially be predictive of future outcome.

There are several limitations of this study. First, due to its retrospective nature and small numbers, we run the risk of type II error in our analysis. Furthermore, PWI was only available in a limited number of our patients, and no gold standard information on collateral status exists for this cohort. In addition, information pertaining to stroke etiology as well as thrombus composition is also lacking in our cohort. In addition, this cohort comprises patients treated with tPA and placebo, and adjustment for treatment group in multivariable regression analyses only partially compensates for this limitation of a heterogeneous cohort. However, this is the first study to investigate the diagnostic and prognostic value of FHVs in a cohort of patients stemming from a multinational, randomized, placebo-controlled trial.

In summary, FHVs may serve as a surrogate marker of large vessel occlusion and successful activation of collaterals to increase blood flow to hypoperfused tissue; in turn, early reduction of FHVs is also an independent predictor of successful recanalization. Although there is no clear clinical relevance for the extent of FHV alone in terms of functional recovery, FHVs may modify treatment effect of thrombolysis. In other words, patients with less pronounced FHVs on acute imaging seem to profit from rtPA more. We maintain that this frequently observed MRI parameter should not guide treatment decisions based on current findings and that a validation in a larger independent cohort is warranted. However, FHVs may serve as an additional piece of information in selecting patients with confirmed vessel occlusion for intravenous thrombolysis or bridging therapy before endovascular treatment.

## Data Availability Statement

The raw data supporting the conclusions of this article will be made available by the authors, without undue reservation.

## Ethics Statement

The studies involving human participants were reviewed and approved by the WAKE-UP trial protocol was approved by the national regulatory authority in each of the six participating countries (Belgium, Denmark, France, Germany, Spain, and United Kingdom). The trial was approved by the respective national or local ethics committees or institutional review boards of all participating centers. Patients or their legal representatives provided written informed consent according to national and local regulations. The main trial was conducted according to the principles laid down in the Declaration of Helsinki in its version of Seoul, 2008; the EU Clinical Trial Directive 2001/20/EC; the Note for Guidance on Good Clinical Practice (CPMP/ICH/135/95 of January 17, 1997); the applicable national drug laws, e.g., German Drug Law (Arzneimittelgesetz, 15. Novelle, AMG); and the GCP-Regulation from August 9, 2004. The patients/participants provided their written informed consent to participate in this study. Written informed consent was obtained from the individual(s) for the publication of any potentially identifiable images or data included in this article.

## Author's Note

Ischemic stroke is one of the leading causes of death and disability worldwide. Rapid administration of tissue plasminogen activator (i.e., thrombolysis) within 4.5 h of symptom onset has been shown to greatly increase the likelihood of achieving a good outcome (functional independence) following an ischemic stroke. However, not all patients benefit from thrombolysis; therefore, there is a continued search for clinical and imaging parameters that can identify those most likely to achieve a good outcome following treatment. Here, we investigated the diagnostic and prognostic value of a frequently observed MRI sign (so-called hyperintense vessels) in acute ischemic stroke patients with unknown time of symptom onset. Previous studies on the topic have yielded contradictory results. Here, we found that although this MRI sign does not necessarily predict stroke progression or outcome, the degree to which this MRI vessel sign is expressed might modify the treatment effect of thrombolysis. This could be of particular importance in an acute clinical setting when selecting patients who are eligible for thrombolysis. Although these results need to be validated in independent cohorts, they take us closer to understanding individual stroke pathology using clinical routine diagnostics.

## Author Contributions

CG and GT conceived and designed the WAKE-UP trial. FB performed the data analysis. SP and JBF were part of the central image reading board. IG and AG performed imaging analysis specific to this analysis. AKu and IG conceived and designed the current *post hoc* analysis. AKu, AG, and IG wrote the first draft of the manuscript. All authors were involved in patient recruitment, interpreted the data, reviewed and edited the manuscript, and approved the final version of the manuscript.

## Conflict of Interest

JBF reports consulting and advisory board fees from BioClinica, Cerevast, Abbvie, AC Immune, Artemida, Brainomix, Biogen, BMS, Daiichi-Sankyo, Guerbet, Ionis Pharmaceuticals, Julius Clinical, Eli Lilly, Tau Rx, and EISAI outside the submitted work. MEn reports grants from Bayer and fees paid to the Charité from Bayer, Boehringer Ingelheim, BMS, Daiichi Sankyo, Amgen, GSK, Sanofi, Covidien, Novartis, and Pfizer, all outside the submitted work. The remaining authors declare that the research was conducted in the absence of any commercial or financial relationships that could be construed as a potential conflict of interest.

## Abbreviations

DWI: diffusion-weighted imaging
FLAIR: fluid-attenuated inversion recovery
FHV: FLAIR hyperintense vessel
MCA: middle cerebral artery
ICA: internal carotid artery
MRI: magnetic resonance imaging
mRS: modified Rankin Scale
NIHSS: National Institutes of Health Stroke Scale (score)
OR: odds ratio
TOF: time-of-flight
PWI: perfusion-weighted imaging.
